# Single-agent gemcitabine in pretreated patients with non-small-cell lung cancer: results of an Argentinean multicentre phase II trial

**DOI:** 10.1038/sj.bjc.6690774

**Published:** 1999-11

**Authors:** M Van Kooten, G Traine, G Cinat, E Cazap, A Zori Comba, H Vicente, S Sena, O Rodriguez Nievas, M Orlando

**Affiliations:** Alexander Fleming Institute, Cramer 1180, Buenos Aires 1426, Argentina; Hospital Israelita, Buenos Aires, Argentina; Instituto Angel H Roffo, Buenos Aires, Argentina; Instituto Estevez, Buenos Aires, Argentina; Hospital Alemán, Buenos Aires, Argentina; Hospital Zubizarrteta, Buenos Aires, Argentina; Hospital Tornú, Buenos Aires, Argentina; Centro Oncologico de Excelencia, Gonnet, Argentina; Hospital Durand, Buenos Aires, Argentina; Hospital Municipal de Oncologia Marie Curie, Buenos Aires, Argentina

**Keywords:** gemcitabine, advanced non-small-cell lung cancer, second-line chemotherapy

## Abstract

The activity and mild toxicity profile of single-agent gemcitabine therapy in untreated (chemonaive) patients with non-small-cell lung cancer (NSCLC) is well documented. This phase II trial was conducted to determine the objective tumour response rate and toxicity profile of single-agent gemcitabine in pretreated patients with NSCLC. Patients with histological evidence of advanced NCSLC stage IIIB or IV; at least one prior chemotherapy regimen including a platinum or taxane analogue; an Eastern Cooperative Oncology Group (ECOG) performance status of 0–2; clinically measurable disease; adequate bone marrow reserve; and adequate renal function; received 1000 mg m^–2^ gemcitabine administered over 30 min on days 1, 8 and 15 of a 28-day cycle defined as 3 weekly treatments followed by 1 week of rest. Twenty-nine patients were evaluated for efficacy and 32 for toxicity. One patient achieved a complete response and five patients had a partial response resulting in a total response rate of 20.6% (95% confidence interval (CI) 6–34). Median response duration was 7 months (range 4–11 months). Twelve (41%) patients reached stable disease after two cycles of therapy and 11 (38%) patients had disease progression. Median progression-free survival time was 3 months and median overall survival time was 5.5 months. Toxicity was generally mild (grades 0–2). Severe (grade 3 or 4) haematological toxicities included grade 3 anaemia in one patient and grade 3 thrombocytopenia in two patients. Severe non-haematological toxicities included one patient each with grade 3 liver transaminase elevations, nausea/vomiting and diarrhoea. This study confirms the activity and safety of single-agent gemcitabine in pretreated patients with advanced NSCLC who are refractory or sensitive to first-line therapy. © 1999 Cancer Research Campaign


*British Journal of Cancer* (1999) **81**(5), 846–849
© 1999 Cancer Research Campaign
Article no. bjoc.1999.0774

### Single-agent gemcitabine in pretreated patients with non-small-cell lung cancer: results of an Argentinean multicentre phase II trial

**M Van Kooten[1], G Traine[2], G Cinat[3], E Cazap[4], A Zori Comba[6], H Vicente[7], S Sena[5], O Rodriguez Nievas[8] and M Orlando[1]**

[1]Alexander Fleming Institute, Cramer 1180, Buenos Aires 1426, Argentina; [2]Hospital Israelita, Buenos Aires, Argentina; [3]Instituto Angel H Roffo, Buenos Aires, Argentina; [4]Instituto Estevez, Buenos Aires, Argentina; [5]Hospital Alemán, Buenos Aires, Argentina; [6]Hospital Zubizarrteta, Buenos Aires, Argentina; [7]Hospital Tornú, Buenos Aires, Argentina; [8]Centro Oncologico de Excelencia, Gonnet, Argentina; [9]Hospital Durand, Buenos Aires, Argentina; [10]Hospital Municipal de Oncologia Marie Curie, Buenos Aires, Argentina

**Summary** The activity and mild toxicity profile of single-agent gemcitabine therapy in untreated (chemonaive) patients with non-small-cell lung cancer (NSCLC) is well documented. This phase II trial was conducted to determine the objective tumour response rate and toxicity profile of single-agent gemcitabine in pretreated patients with NSCLC. Patients with histological evidence of advanced NCSLC stage IIIB or IV; at least one prior chemotherapy regimen including a platinum or taxane analogue; an Eastern Cooperative Oncology Group (ECOG) performance status of 0–2; clinically measurable disease; adequate bone marrow reserve; and adequate renal function; received 1000 mg $m^{-2}$ gemcitabine administered over 30 min on days 1, 8 and 15 of a 28-day cycle defined as 3 weekly treatments followed by 1 week of rest. Twenty-nine patients were evaluated for efficacy and 32 for toxicity. One patient achieved a complete response and five patients had a partial response resulting in a total response rate of 20.6% (95% confidence interval (CI) 6–34). Median response duration was 7 months (range 4–11 months). Twelve (41%) patients reached stable disease after two cycles of therapy and 11 (38%) patients had disease progression. Median progression-free survival time was 3 months and median overall survival time was 5.5 months. Toxicity was generally mild (grades 0–2). Severe (grade 3 or 4) haematological toxicities included grade 3 anaemia in one patient and grade 3 thrombocytopenia in two patients. Severe non-haematological toxicities included one patient each with grade 3 liver transaminase elevations, nausea/vomiting and diarrhoea. This study confirms the activity and safety of single-agent gemcitabine in pretreated patients with advanced NSCLC who are refractory or sensitive to first-line therapy. © 1999 Cancer Research Campaign

**Keywords:** gemcitabine; advanced non-small-cell lung cancer; second-line chemotherapy

Although many cytotoxic drugs have been tested as single agents in patients with advanced non-small-cell lung cancer (NSCLC), only a few (cisplatin, vindesine, mitomycin, ifosfamide, vinblastine, irinotecan and taxanes) have produced response rates greater than 15% (Ihde, 1992). Currently, cisplatin is a standard agent used in combination therapies for NSCLC. Results of a randomized trial (Rapp et al, 1988) and a recent meta-analysis from 52 randomized trials demonstrated that cisplatin-containing regimens improve, albeit modestly, survival benefit compared to best supportive care in patients with advanced NSCLC (NSCLC Collaborative Group, 1995). New agents that have become available in the 1990s have consistently demonstrated significant anti-tumour activity and encouraging toxicity profiles, while incorporating different mechanisms of anti-tumour action. One such agent, gemcitabine, is an antimetabolite structurally similar to cytarabine (ARA-C), but possesses a unique mechanism of action that exerts a much wider range of anti-tumour activity in vitro (Hertel et al, 1990; Lund et al, 1993). Gemcitabine mimics the structure of the naturally occurring nucleoside, deoxycytidine, and thus is inserted into the nucleoside sites of DNA. The additional nucleoside in the DNA strand masks gemcitabine from DNA repair mechanisms that might excise it. This 'masked chain' effect allows gemcitabine to exert a wide spectrum of anti-tumour activity against human neoplasms, such as lung, ovarian, and pancreatic cancers (Gatzemeier et al, 1996; Rothenberg et al, 1996).

In phase II trials performed in chemo-naïve patients with NSCLC, first-line treatment with gemcitabine (1000–1250 mg $m^{-2}$) produced consistent and reproducible response rates of approximately 20–23% (Abratt et al, 1994; Anderson et al, 1994; Gatzemeier et al, 1996). In all of these studies, which used the gemcitabine weekly regimen (intravenous infusion over 30 min given weekly for 3 weeks every 28 days), the toxicity profile was modest and characterized by mild leukopenia and thrombocytopenia, and other negligible toxic effects, making it an acceptable choice for combination therapy. In addition, response rates and toxicity profiles of gemcitabine are similar in pretreated and untreated patients with pancreatic cancer (Rothenberg et al, 1996) and lung cancer (Crinó et al, 1997*a*). This phase II trial was conducted to determine the objective tumour response rate and

*Received 29 January 1999*
*Revised 10 May 1999*
*Accepted 7 June 1999*

*Correspondence to:* M Orlando

**Table 1**

| Leucocytes × mm³ | | Platelets × mm³ | Dose given |
|---|---|---|---|
| > 3.000 | y | > 100.000 | 100% |
| 1.500–3.000 | ó | 50.000–100.000 | 75% |
| < 1.500 | ó | < 50.000 | Hold |

toxicity profile of gemcitabine used as a single agent in pretreated patients with NSCLC.

### PATIENTS AND METHODS

The protocol was approved by the local Ethics Committees at participating centres and patients signed informed consent prior to inclusion. Patients were included in the study if they had histological evidence of advanced stage IIIb or IV not amenable to curative surgery or radiation; at least one prior chemotherapy regimen including a platinum or taxane analogue; an Eastern Cooperative Oncology Group (ECOG) performance status of 0–2; clinically measurable disease defined as bidimensionally measurable lesions; adequate bone marrow reserves; and adequate hepatic and renal function. Patients received 1000 mg $m^{-2}$ gemcitabine administered by intravenous infusion over 30 min on days 1, 8 and 15 of a 28-day cycle defined as 3 weeks of treatment followed by 1 week of rest. Treatment was continued until progressive disease or unacceptable toxicity occurred with no maximum number of cycles imposed on the patients. Premedication and prophylactic antiemetic therapy was left to the discretion of the investigator. Dose adjustments and omissions were scheduled for patients experiencing grade 3 or 4 haematological toxicities according to the guidelines shown in Table 1.

All patients who received at least one cycle of gemcitabine and met protocol criteria were included in the efficacy analyses. All patients who received at least one gemcitabine dose and met all protocol entry criteria were included in the safety analyses. Survival was measured from the day of the first dose until the day of death. Progression-free survival was measured from the first day of treatment until the day of progressive disease or discontinuation of treatment. Objective tumour response rates and survival times were computed and survival curves were generated using the Kaplan–Meier method. Toxicity and tumour response were assessed using World Health Organization (WHO) criteria.

### RESULTS

A total of 34 patients were registered and 33 patients entered the study and received gemcitabine at 13 Argentinean centres between December 1996 and Februrary 1998 (one patient withdrew from protocol before the start of gemcitabine and was lost to follow-up). Thirty-two patients (23 males and nine females) with a median age of 58 years were included in the analyses (32 evaluable for toxicity and 29 for efficacy). Among these patients, all had stage IIIb (15 patients) or stage IV (17 patients) disease, and most had histological evidence of adenocarcinoma (20 patients) and received at least one prior chemotherapy regimen containing a platinum or taxane analogue. Patients' characteristics are presented in Table 2. Patients received a total of 102 cycles with a median of 3 cycles per patient (range 1–8 cycles).

**Table 2** Patient characteristics

| Characteristic | |
|---|---|
| Total registered | 34 |
| Total entered (received drug) | 33 |
| Total evaluable | 32 |
| Efficacy | 29 |
| Safety | 32 |
| Number males | 23 |
| Number females | 9 |
| Median age | 58 |
| ECOG performance status: | |
| 0 | 5 |
| 1 | 18 |
| 2 | 5 |
| Unspecified | 4 |
| Stage | |
| Recurrent IIIB | 15 |
| IV | 17 |
| Histology | |
| Adenocarcinoma | 20 |
| Squamous | 9 |
| Large cell | 3 |
| Evaluable disease pattern | |
| Lung | 25 |
| Nodes | 9 |
| Liver | 6 |
| Adrenal | 3 |
| Bone | 2 |
| Skin | 2 |
| Prior therapy | |
| Cisplatin/carboplatin + etoposide | 13 |
| Cisplatin/carboplatin + paclitaxel | 8 |
| Cisplatin + vinorelbine | 4 |
| Other cisplatin combinations | 2 |
| Taxanes (single agents) | 2 |
| Other taxane combinations | 5 |
| Radiotherapy | 5 |

ECOG = Eastern Cooperative Oncology Group.

**Table 3** Response rate

| | |
|---|---|
| Patients evaluable | 29 |
| Complete response | 1 (3.4%) |
| Partial response | 5 (17.2%) |
| Total objective response rate | 20.6% |
| Stable disease | 12 (41.4%) |
| Progressive disease | 11 (37.9%) |

Of the 33 patients who entered the study and actually received the drug, four were considered ineligible for the efficacy analyses: three patients had prior radiotherapy on the only site of measurable disease, and one patient received concomitant treatment with carboplatin. This last patient was also excluded from toxicity analysis. Response rates are presented in Table 3. One patient achieved a complete response (adrenal metastasis as the only site of disease, in a patient progressing after prior chemotherapy with carboplatin+etoposide) and five patients had a partial response resulting in a total response rate of 20.6% (95% confidence interval (CI) 6–34). The median response duration was 7 months (range 4–11 months). Twelve (41%) patients reached stable disease after two cycles of therapy and 11 (38%) patients had disease progression; disease progression occurred in the first cycle for two patients. External validation of claimed responses was not performed.

*© 1999 Cancer Research Campaign*

**Table 4** Chemotherapy sensitivity of responders ($n = 6$)

| Patient number | Initial regimen | Response to 1st line | Response to Gemcitabine |
|---|---|---|---|
| 1 | Docetaxel | Complete response | PR |
| 2 | Cisplatin/Paclitaxel | Minor response | PR |
| 3 | Cisplatin/Etoposide | Stable disease | PR |
| 4 | Carboplatin/Etoposide (adjuvant) | Relapse within 2 months | PR |
| 5 | Carboplatin/Paclitaxel | Progressive disease | PR |
| 6 | Carboplatin/Etoposide | Progressive disease | CR |

Responders represented patients across a wide age group (56–76 years), a range of performance levels (0–2) and a range of prior chemotherapy regimens including single-agent docetaxel in one patient, platinum/etoposide in three patients and carboplatin/paclitaxel combination in two patients (Table 4). All but one of the responders had adenocarcinoma. The disease among responders was predominantly in the lung and mediastinal nodes, but was also present in the adrenal gland (two patients) and the liver (metastasis in one patient).

Survival curves are shown in Figure 1. Median progression-free survival time was 3 months (95% CI 2.7–5.4 months) and median overall survival time was 5.5 months (95% CI 4.2–7.3 months). Survival curves show 43% of patients alive at 6 months and 29% alive at 1 year. Twenty-four patients had died as of June 1998.

One of the patients excluded from the efficacy analyses because of concomitant treatment with carboplatin, was also excluded from the toxicity analyses. Thus, 32 patients were evaluated for toxicity. Toxicity was generally mild (grades 0–2). Laboratory and non-laboratory toxicities are presented in Tables 5 and 6 respectively. Severe (grade 3 or 4) haematological toxicities included grade 3 anaemia in one patient and grade 3 thrombocytopenia in two patients. Severe non-haematologic toxicities included one patient each with grade 3 liver transaminase elevations, nausea/vomiting and diarrhoea. Clinically significant asthenia was noted in three patients. Eleven doses (out of 306 planned injections) were omitted, ten doses were reduced and seven doses were delayed primarily due to leukopenia and thrombocytopenia toxicities.

**Table 5** WHO haematologic toxicity number (%) of patients

| | **Maximum WHO grade attained $n = 32$** | | | |
|---|---|---|---|---|
| **Toxicity** | **0–1** | **2** | **3** | **4** |
| Anaemia | 29 (91) | 2 (6) | 1 (3) | 0 |
| Leukopenia | 28 (88) | 4 (13) | 0 | 0 |
| Thrombocytopenia | 28 (88) | 2 (6) | 2 (6) | 0 |

WHO = World Health Organization.

**Table 6** WHO non-haematologic toxicity Number (%) of patients

| | **Maximum WHO grade attained $n = 32$** | | | |
|---|---|---|---|---|
| **Toxicity** | **0–1** | **2** | **3** | **4** |
| Liver transaminases[a] | 30 (94) | 1 (3) | 1 (3) | 0 |
| Creatinine | 32 (100) | 0 | 0 | 0 |
| Alopecia | 31 (97) | 1 (3) | 0 | 0 |
| Nausea/vomiting | 30 (94) | 1 (3) | 1 (3) | 0 |
| Skin rash | 30 (94) | 2 (6) | 0 | 0 |
| Peripheral oedema | 32 (100) | 0 | 0 | 0 |
| Dyspnoea/chest pain | 32 (100) | 0 | 0 | 0 |
| Asthenia | 29 (91) | 3 (9) | 0 | 0 |
| Phlebitis | 30 (94) | 2 (6) | 0 | 0 |
| Diarrhoea | 30 (94) | 1 (3) | 1 (3) | 0 |
| Mucositis | 31 (97) | 1 (3) | 0 | 0 |

WHO = World Health Organization. [a] Alanine aminotransferase (ALT) and aspartate aminotransferase (AST).

### DISCUSSION

Gemcitabine is an active agent against advanced NSCLC when used alone or as part of a combination regimen. In this study and in other studies in pretreated patients (Crinó et al, 1997*a*; Guerra et al, 1997; Piazza et al, 1997; Rosvold et al, 1998), single-agent gemcitabine as second-line therapy produced an overall response rate of approximately 20%, which is similar to response rates observed in single-agent gemcitabine studies in untreated patients, who are not cross-resistant with other agents commonly used in

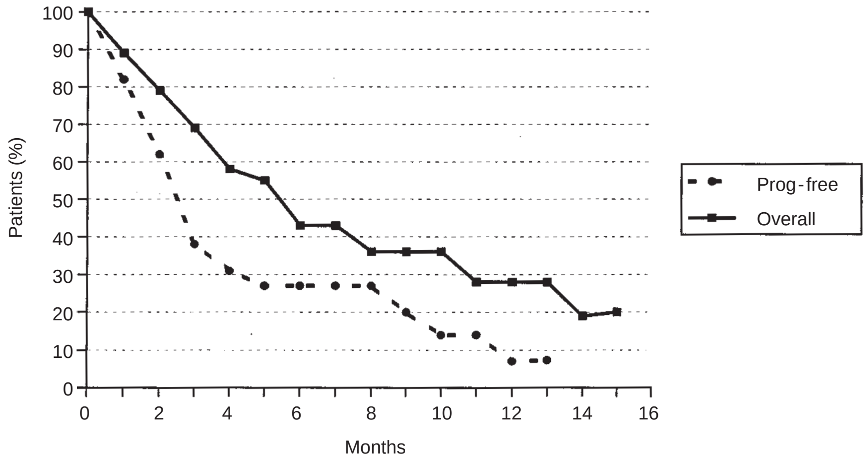

**Figure 1** Gemcitabine in pretreated patients with NSCLC. Progression-free and overall survival

*© 1999 Cancer Research Campaign*

this setting. Given an appropriate regimen, gemcitabine is considered to interact synergistically with cisplatin to enhance its cytotoxicity as evidenced by the clinical results observed in first-line treatment with gemcitabine–cisplatin combination regimens producing response rates of 30–50% (Abratt et al, 1997; Crinó et al, 1997*b*; Einhorn, 1997; Shepherd et al, 1997). In patients who are unable to tolerate the greater toxicity associated with cisplatin-based therapies, who are either sensitive (prior responders) or refractory (prior non-responders) to first-line therapy, single-agent gemcitabine with its proven activity and modest side-effects offers an alternative to combination therapy. In this study, patients with advanced disease who were refractory or sensitive to first-line therapy (including taxane and platinum analogues) (Table 4), and who represented a wide age group (56–76 years), a range of performance levels (0–2), and a range of prior chemotherapy regimens had excellent response rates and tolerable toxicities to gemcitabine therapy. In addition, the toxicity profile in this study was similar to those observed in other single-agent gemcitabine studies in untreated patients with NSCLC, as was previously reported by Crinó et al (1997*a*) and Rothenberg et al (1996) for second-line lung and pancreas cancer patients.

Although not formally evaluated in this study, symptomatic improvement might be as important an end point as objective response in this patient population. In a single-agent gemcitabine study in pretreated patients with NSCLC, symptomatic benefit was evaluated and reported as significant (Guerra et al, 1997), although the value of gemcitabine as second-line therapy compared to that of best supportive care has yet to be determined in a randomized trial in pretreated patients.

Given the fact that the benefits of first-line platinum-based chemotherapy are modest, indications for second-line therapy are even more arguable. However, increasing numbers of patients with relapsed NSCLC, but otherwise in good condition (PS 0–1), seek second-line therapy, even in the absence of proven benefit.

Few agents have shown consistent activity in the setting of platinum-pretreated NSCLC, docetaxel being the most active so far studied (Fossella et al, 1997); other agents reported reveal contradictory data.

Gemcitabine, based on the available data, shows at least a degree of activity similar to docetaxel and might prove useful also in the presence of taxane-resistant NSCLC. One of the potential advantages of gemcitabine would be represented by its minimal general toxicity, and in particular, the absence of overlapping toxicities with platinum and taxanes such as neurotoxicity and alopecia.

In conclusion, this study confirms the activity of gemcitabine administered as a single agent in pretreated patients. The effectiveness and mild toxicity profile of gemcitabine encourages its use in patients with locally advanced or metastatic NSCLC as single therapy or in combination with other agents in first-line treatment and as a single agent for second-line patients who relapse or progress after platinum- or taxane-based chemotherapy.

### ACKNOWLEDGEMENTS

This trial was supported in part by a grant from Eli Lilly. Investigators and Participating Centers – Maximiliano Van Kooten MD, Mauro Orlando MD: Alexander Fleming Institute, Buenos Aires; Gabriel Traine MC, Simón Breier MD: Hospital Israelita, Buenos Aires; Gabriela Cinat MD, Elisabeth Mickiewicz MD, Celia Brosio MD: Instituto Angel H. Roffo, Buenos Aires; Eduardo Cazap MD, Jeannette Roger MD: Instituto Estevez, Buenos Aires; Susana Sena MD, Federico Coppola MD: Hospital Alemán, Buenos Aires; Alberto Zori Comba MD: Hospital Zubizarrteta, Buenos Aires; Claudia Bagnes MD, Héctor Vicente MD: Hospital Tornú, Buenos Aires; Ofelia Rodriguez Nievas MD, Alberto Luchina MD: Centro Oncologico de Excelencia, Gonnet; Alberto Goldfarb, Leonardo Koliren MD: Hospital Durand, Buenos Aires; Raquel Oliva MD: Hospital Municipal de Oncologia Marie Curie, Buenos Aires.

### REFERENCES

Abratt RP, Bezwoda WR, Falkson G, et al (1994) Efficacy and safety profile of gemcitabine in non-small cell lung cancer: a phase II study. *J Clin Oncol* **12**: 1535–1540

Abratt RP, Bezwoda WR, Goedhale L, et al (1997) Weekly gemcitabine with monthly cisplatin: effective chemotherapy for advanced non-small cell lung cancer. *J Clin Oncol* **15**: 744–749

Anderson H, Lund B, Bach F, et al (1994) Single agent activity of weekly gemcitabine in advanced non-small cell lung cancer: a phase II study. *J Clin Oncol* **12**: 1821–1826

Crinó L, Mosconi A, Scagliotti G, et al (1997*a*) Salvage therapy in pretreated, advanced non-small-cell lung cancer. *Proc Am Soc Clin Oncol* **17**: 1603 (Abstract)

Crino L, Scagliotti G, Marangolo M, et al (1997*b*) Cisplatin-gemcitabine combination in advanced non-small cell lung cancer. *Proc Am Soc Clin Oncol*: **15**: 297–303

Einhorn L (1997) Phase II trial of gemcitabine plus cisplatin in non-small cell lung cancer. Hoosier Oncology Group Study. *Semin Oncol* **24**: S8 24–S8 26

Fossella F, Lee JS and Hong WK (1997) Management strategies for recurrent non-small cell lung cancer. *Semin Oncol* **24**: 455–462

Gatzemier U, Shepherd, FA, Le-Chevalier T, et al (1996) Activity of gemcitabine in patients with non-small cell lung cancer: a multicenter extended phase II study. *Eur J Cancer* **32**: 243–248

Guerra J, Lianes P, Paz-Ares L, et al (1997) Efficacy and toxicity profile of gemcitabine in previously treated patients with non-small cell lung cancer. Lung Cancer (suppl) World Conference on Lung Cancer, Dublin (abstract no. 99).

Hertel LW, Boder GB, Kroin, JS, et al (1990) Evaluation of the antitumor activity of gemcitabine (2′,2′-difluoro-2′-deoxycytidine). *Cancer Res* **50**: 4417–4422

Ihde DC (1992) Chemotherapy of lung cancer. *N Engl J Med* **327**: 1434–1441

Lund B, Kristjansen PEG, Hansen HH (1993) Clinical and preclinical activity of 2′,2′- difluoro-2′-difluorodeoxycytidine (gemcitabine). *Cancer Treat Rev* **19**: 45–55

Non-small Cell Lung Cancer Collaborative Group (1995) Chemotherapy in non-small cell lung cancer: a meta-analysis using updated data on individual patients from 52 randomized clinical trials. *Br Med J* **311**: 899–909

Piazza E, Isa L, Pavia GF, et al (1997) Gemcitabine in the treatment of pretreated metastatic non-small cell lung cancer: a preliminary report. Lung Cancer (suppl) World Conference on Lung Cancer, Dublin (Abstract no 138).

Rapp E, Parer IL, Willan A, et al (1988) Chemotherapy can prolong survival in patents with advanced non-small cell lung cancer: report of a Canadian multicenter randomized trial. *J Clin Oncol* **6**: 633–641

Rosvold E, Langer CJ, Schelder R, et al (1998) Salvage therapy with gemcitabine in advanced non-small cell lung cancer progressing after prior carboplatin-paclitaxel. *Proc Am Soc Clin Oncol* **12**: 1797

Rothenberg ML, Moore MJ, Cripps MC, et al (1996) A phase II trial of gemcitabine in patients with 5-FU refractory pancreas cancer. *Ann Oncol* **7**: 347–352

Shepherd F, Cormier Y, Burkes R, et al (1997) Phase II trial study of gemcitabine and weekly cisplatin for advanced non-small cell lung cancer. *Semin Oncol* **24** (suppl 8): S27–S30

Word Health Organization (1979) *WHO Handbook for Reporting Results of Cancer Treatment*, p. 48. WHO: Geneva

*© 1999 Cancer Research Campaign*
